# Radiomics-based prediction of recurrent acute pancreatitis in individuals with metabolic syndrome using T2WI magnetic resonance imaging data

**DOI:** 10.3389/fmed.2025.1502315

**Published:** 2025-03-06

**Authors:** Yuan Wang, Xiyao Wan, Ziyan Liu, Ziyi Liu, Xiaohua Huang

**Affiliations:** Department of Radiology, Affiliated Hospital of North Sichuan Medical College, Nanchong, China

**Keywords:** acute pancreatitis, metabolic syndrome, recurrence, magnetic resonance imaging, radiomics

## Abstract

**Objective:**

This study sought to clarify the utility of T2-weighted imaging (T2WI)-based radiomics to predict the recurrence of acute pancreatitis (AP) in subjects with metabolic syndrome (MetS).

**Methods:**

Data from 196 patients with both AP and MetS from our hospital were retrospectively analyzed. These patients were separated into two groups according to their clinical follow-up outcomes, including those with first-onset AP (*n* = 114) and those with recurrent AP (RAP) (*n* = 82). The 196 cases were randomly divided into a training set (*n* = 137) and a test set (*n* = 59) at a 7:3 ratio. The clinical characteristics of these patients were systematically compiled for further analysis. For each case, the pancreatic parenchyma was manually delineated slice by slice using 3D Slicer software, and the appropriate radiomics characteristics were retrieved. The K-best approach, the least absolute shrinkage and selection operator (LASSO) algorithm, and variance thresholding were all used in the feature selection process. The establishment of clinical, radiomics, and combined models for forecasting AP recurrence in patients with MetS was then done using a random forest classifier. Model performance was measured using the area under the receiver operating characteristic curve (AUC), and model comparison was done using the DeLong test. The clinical utility of these models was evaluated using decision curve analysis (DCA), and the optimal model was determined via a calibration curve.

**Results:**

In the training set, the clinical, radiomics, and combined models yielded respective AUCs of 0.651, 0.825, and 0.883, with corresponding test sets of AUCs of 0.606, 0.776, and 0.878. Both the radiomics and combined models exhibited superior predictive effectiveness compared to the clinical model in both the training (*p* = 0.001, *p* < 0.001) and test sets (*p* = 0.04, *p* < 0.001). The combined model outperformed the radiomics model (training set: *p* = 0.025, test set: *p* = 0.019). The DCA demonstrated that the radiomics and combined models had greater clinical efficacy than the clinical model. The calibration curve for the combined model demonstrated good agreement between the predicted probability of AP recurrence and the observed outcomes.

**Conclusion:**

These findings highlight the superior predictive power of a T2WI-based radiomics model for predicting AP recurrence in patients with MetS, potentially supporting early interventions that can mitigate or alleviate RAP.

## 1 Introduction

Acute pancreatitis (AP) affects 30–40 per 100,000 people annually and is one of the most common gastrointestinal problems to be diagnosed (1–3). The condition, which is characterized by inflammatory pancreatic cell infiltration, typically presents with symptoms that include pyrexia, dyspepsia, and severe abdominal pain (4). While symptom control can be achieved in most patients within a reasonably short interval, recurrent AP (RAP) can develop in 17–35% of cases (5). Such recurrence tends to coincide with a worse overall patient condition, together with a greater risk of chronic pancreatic damage and dysfunction, as well as higher odds of future pancreatic oncogenesis, which can ultimately lead to a reduced patient survival rate (6–8).

Several metabolic issues, such as obesity, decreased levels of high-density lipoprotein cholesterol (HDL-C), hypertension, hypertriglyceridemia, and hyperglycemia, are indicative of metabolic syndrome (MetS) (9). MetS affects an estimated 25% of people worldwide and is associated with an increased risk of type 2 diabetes, coronary heart disease, stroke, and all-cause death (10, 11). Research has shown that insulin resistance, fatty acid flux and chronic low-grade inflammation are key mechanisms in the pathogenesis of MetS. Among these, insulin resistance is a core mechanism, with its severity driven by excessive fatty acids resulting from inappropriate lipolysis (12). When free fatty acids (FFAs) exceed the normal binding capacity of albumin, they may directly damage pancreatic acinar cells and capillary endothelial cells, leading to RAP (13). Excess FFAs also accumulate in pancreatic capillaries, impairing blood supply and causing thrombus formation in the pancreatic microcirculation. This triggers may repeat ischemic necrosis of the pancreas, ultimately leading to RAP (14). Moreover, as MetS is a chronic low-grade inflammatory state, inflammatory markers such as IL-6 and TNF-α are elevated, and these inflammatory factors may exacerbate pancreatic inflammation and increase the risk of AP recurrence (15). Furthermore, studies have shown that patients with MetS tend to experience more severe AP, higher rates of local and systemic complications, prolonged hospitalization, and higher rates of death (9, 16). AP recurrence has also been closely linked to specific components of MetS, including obesity, diabetes, and hypertriglyceridemia (17–19). These prior findings, however, have been based on the clinical characteristics of affected patients without any corresponding investigation of the underlying functional and structural changes in organs that coincide with disease incidence, failing to fully encapsulate the heterogeneous nature of the biological and pathological features associated with the condition. There have also been few imaging studies focused on RAP in patients with MetS.

Radiomics approaches can enable the extraction of quantitative features from imaging data that are not readily apparent to the naked eye but can effectively capture disease-related heterogeneity (20). Previous studies have demonstrated that radiomics exhibits excellent performance in the diagnosis of pancreatitis and the prediction of its onset and progression (2, 21–23). Magnetic resonance imaging (MRI), with its superior soft tissue resolution, is now considered the first-line imaging modality for evaluating pancreatitis, especially with T2-weighted imaging (T2WI), which is highly sensitive to fluid accumulation and mild peripancreatic inflammation (24, 25). Compared to contrast-enhanced MRI, T2WI does not require contrast agents and is not affected by perfusion changes, which may lead to variability in radiomics feature extraction. Additionally, the T2WI used in this study employs a long repetition time (TR) scan, offering a high signal-to-noise ratio, which allows for the capture of the micro-heterogeneity of pancreatic tissue, providing a reliable foundation for radiomics feature extraction. As such, this study was developed to construct a T2WI radiomics-based model suitable for the quantitative prediction of AP recurrence in individuals with MetS, thereby aiding clinicians in identifying AP patients who face a greater risk of recurrence so that timely interventions can be applied to mitigate such risk.

## 2 Materials and methods

### 2.1 Study subjects

Clinical and imaging data from AP patients with MetS treated from June 2021 through March 2024 was retrospectively analyzed. Ethical permission for this investigation was granted, and informed consent was not required.

AP diagnoses were made based on the 2012 Atlanta criteria (26): (1) Abdominal pain with the characteristics expected for pancreatitis; (2) Serum amylase or lipase levels exceeding the upper limit of normal by at least three times; and (3) Abdominal imaging findings consistent with AP-related findings.

RAP was defined for this study based on the following (27): (1) A history of at least two episodes of AP; (2) An interval of >3 months between AP episodes; and (3) Patients had achieved recovery or near-total recovery during the period between AP episodes.

At least three of the following factors were required for the diagnosis of MetS to be made (28): (1) Obesity, as determined based on national and population-specific waist circumference values; (2) hyperglycemia, as determined by a history of diagnosed and treated diabetes or a fasting blood glucose level ≥ 100 mg/dL (5.55 mmol/L); (3) hypertension, as determined by a history of prior diagnosis and antihypertensive treatment or systolic/diastolic blood pressure ≥ 130/85 mmHg; (4) Triglyceride levels ≥150 mg/dL (1.7 mmol/L); and (5) HDL-C < 40 mg/dL (1.03 mmol/L) or < 50 mg/dL (1.29 mmol/L) for males and females, respectively. Because this study was retrospective and there were no regular measurements of waist circumference among inpatients, participants were considered obese if their BMI was greater than 28 kg/m^2^ (29).

Patients were excluded from this study if they exhibited: (1) AP but not MetS; (2) any concurrent benign or malignant pancreatic tumors; (3) episodes of acutely exacerbated chronic pancreatitis; (4) missing clinical data, loss to follow-up, or poor quality images; or (5) an age < 18 years.

**Figure 1:**
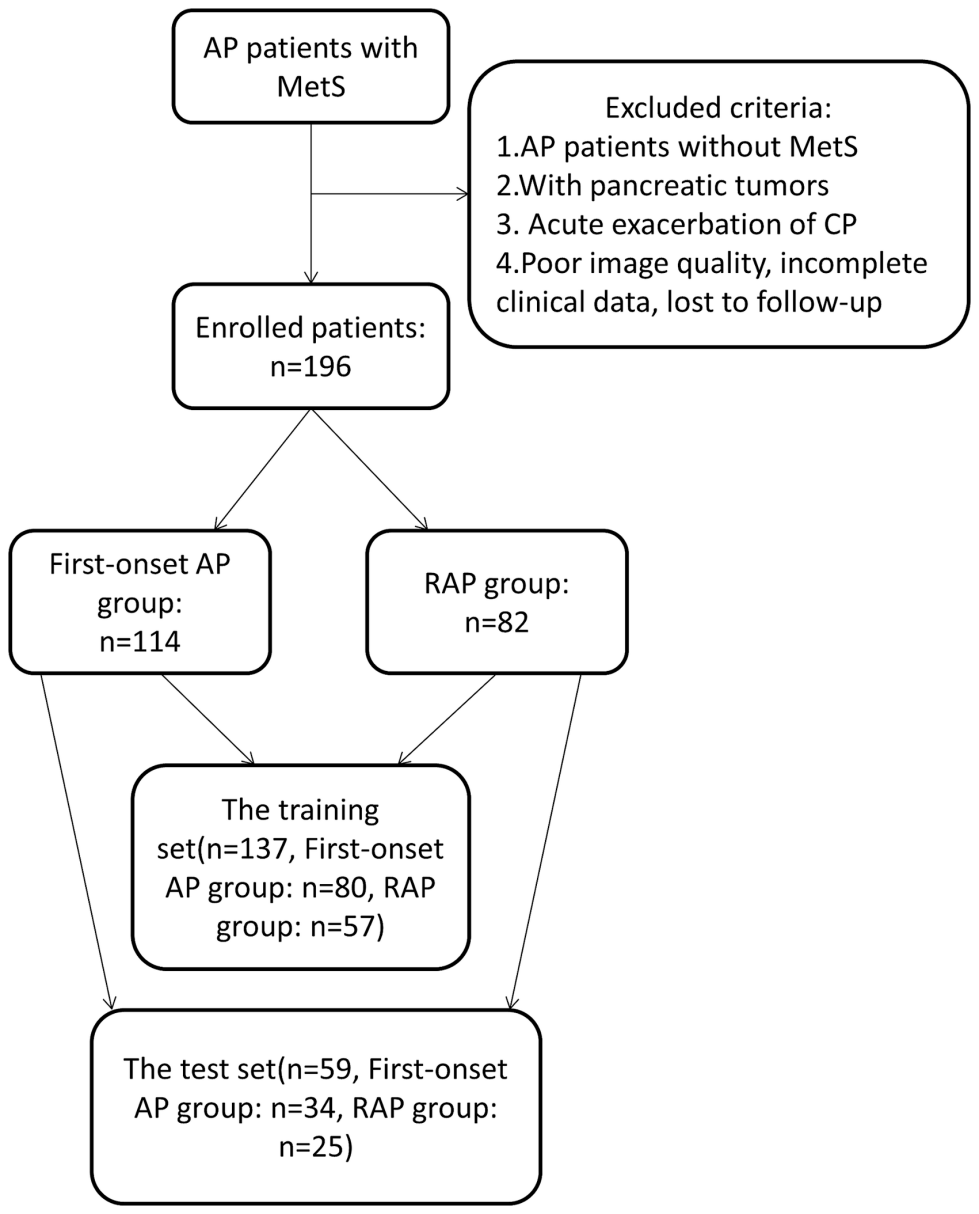
Flow chart of patient recruitment in this study.

This study included 196 AP patients with MetS, of whom 114 were first-onset AP cases and 82 had RAP. The patients were randomized into two sets: a training set (*n* = 137) and a test set (*n* = 59) at a ratio of 7:3 (Figure 1).

### 2.2 Scanning protocol

A 16-channel phased-array coil MRI instrument (Unite Imaging Healthcare, uMR790) was used for plain upper abdominal scanning for all patients. Before scanning, patients were directed to refrain from eating and drinking and were provided respiratory training. Scanning was conducted supine with head entry, placing a respiratory gating device at maximal abdominal movement. The entire upper abdomen was scanned during the procedure, and an axial rapid spin-echo was carried out using the following settings: 8,000 ms for TR; 116 ms for echo time; 38 cm × 30 cm for field of view; 5.0 mm for slice thickness; 2.0 mm for slice gap; and 256 × 171 for matrix size.

### 2.3 Image segmentation and feature extraction

**Figure 2:**
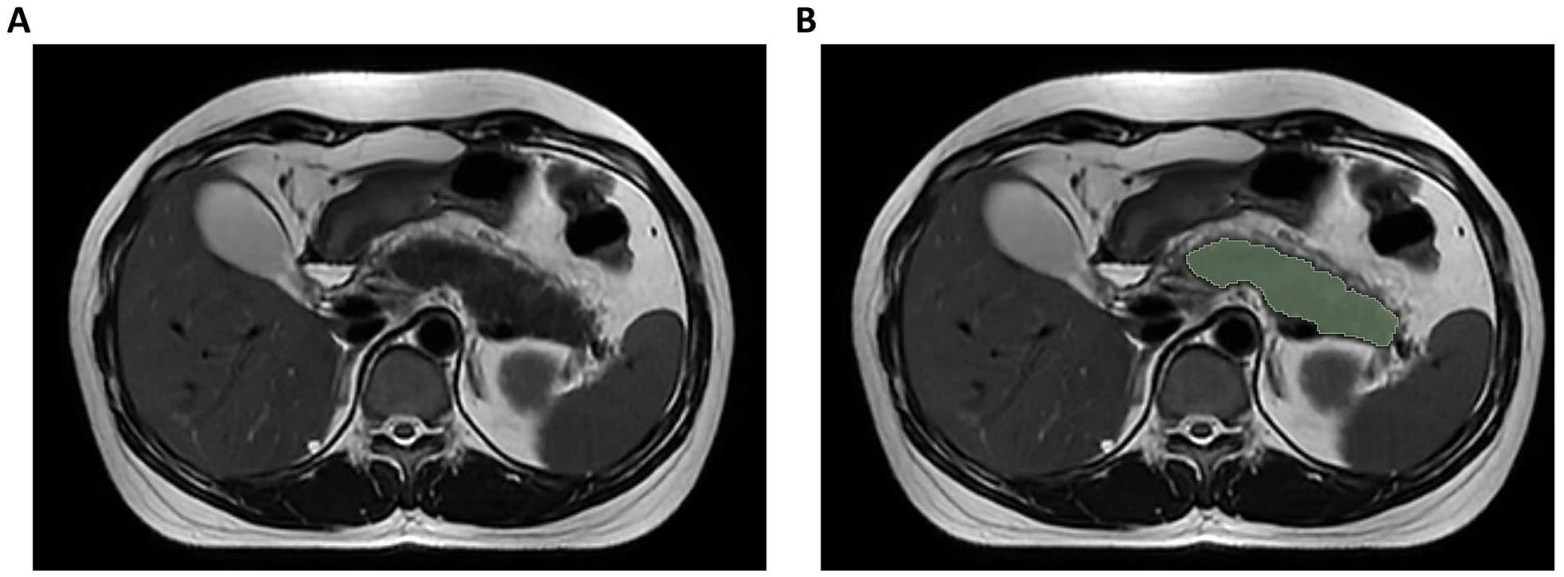
Illustration of axial T2-weighted MRI image segmentation. **(A)** Original image; **(B)** Delineation of region of interest.

Pancreatic segmentation was performed manually on T2WI images using 3D Slicer software (v5.2.2^1^) by an experienced radiologist. The pancreatic parenchyma was carefully delineated slice by slice while ensuring the exclusion of surrounding structures such as the intestines, blood vessels, and the common bile duct. When the pancreatic tissue boundaries were unclear in severe cases, T1-weighted and contrast-enhanced images were referenced to improve segmentation accuracy. In cases where necrosis extended into the peri-pancreatic region, only the pancreatic component was included in the region of interest (ROI), while extra-pancreatic necrotic areas were excluded (Figure 2). After applying the Laplacian of Gaussian and wavelet filtering to the original images, 1,223 features were obtained, including shape features, neighborhood gray-tone difference matrix (NGTDM), gray-level size zone matrix (GLSZM), gray-level run-length matrix (GLRLM), gray-level dependence matrix (GLDM), gray-level co-occurrence matrix (GLCM), and first-order features. Of the analyzed patients, one-third were selected randomly, and the target ROI was independently delineated by another senior radiologist blinded to patient clinical data. The interclass correlation coefficient (ICC) was then used to assess inter-observer reliability, with an ICC > 0.75 deemed reliable such that all features clearing this threshold were retained for further analysis.

### 2.4 Feature selection and ranking

Those features exhibiting an ICC > 0.75 were normalized to eliminate differences in dimensionality among these features through Z-score preprocessing. A multi-step feature selection process was employed to ensure the robustness and generalizability of the model. Initially, variance thresholding was used to remove features with low variability (<0.8). The K-best method was then applied to rank features based on their statistical association with AP recurrence, retaining the most discriminative ones. Subsequently, the least absolute shrinkage and selection operator (LASSO) regression was implemented to eliminate redundant features and select the most informative predictors. This sequential integration of methods helped enhance model stability and avoid overfitting. The Gini impurity-reduction feature-ranking technique was then used to rank the final sets of radiomics and clinical features linked with AP recurrence based on the random forest algorithm.

### 2.5 Model development and assessment

Significant clinical features and optimal radiomics features were used to develop clinical and radiomics models through a random forest classifier, with a combined model integrating both feature sets also being established. Using criteria including the area under the receiver operating characteristic (ROC) curve (AUC), sensitivity, specificity, and accuracy, these models’ ability to forecast AP recurrence in MetS patients was assessed. While the clinical value of these models was investigated using a decision curve analysis (DCA), model predictive efficacy was evaluated in line with the DeLong test. A calibration curve was constructed for the combined model. R (v4.3.2^2^) and the United Imaging uAI Research Portal (v1.6) were used for feature selection and model generation.

### 2.6 Statistical analyses

All analyses were conducted using SPSS 26.0, with normally distributed data given as means ± standard deviation and skewed data as median (interquartile range). Comparisons were made using independent sample *t*-tests and Mann–Whitney *U* tests. Categorical data were reported as numbers (%) and compared with χ^2^ tests. Risk variables independently associated with AP recurrence in individuals with MetS were identified using multivariate logistic regression analysis, with *p* < 0.05 indicating statistical significance.

## 3 Results

### 3.1 Clinical data

Table 1 summarizes the clinical characteristics of the study participants. No additional significant changes were observed between the first-onset AP and RAP groups, but there were substantial differences in the levels of triglycerides, total cholesterol, and hyperlipidemia (*p* < 0.05). Triglyceride levels were found to be independently associated with the probability of AP recurrence in patients with MetS in multivariate logistic regression analysis, with an odds ratio (OR) of 1.061 (95% confidence interval [CI]: 1.021–1.102).

**Table 1:** Clinical characteristics of patients with AP and MetS.

Characteristics	First-onset AP group (*n* = 114)	RAP group (*n* = 82)	*p* value
Age (years)	48 (36–56)	46 (37–53)	0.290
Sex (male/female)	67/47	47/35	0.839
BMI (kg/m^2^)	26.9 (24.3–29.3)	26.6 (24.4–28.9)	0.756
Hypertension (*n*/%)	27/23.7	18/22.0	0.776
Hyperlipidemia (*n*/%)	77/67.5	66/80.5	0.044
Biliary stones (*n*/%)	39/34.2	16/19.5	0.330
Diabetes mellitus (*n*/%)	36/31.6	27/32.9	0.842
Total cholesterol (mmol/L)	5.0 (4.5–6.4)	6.0 (4.8–8.2)	0.004
Triglyceride (mmol/L)	3.7 (2.0–8.5)	6.6 (3.4–14.2)	0.001
HDL-C (mmol/L)	0.8 (0.7–1.0)	0.7 (0.6–0.9)	0.055
Smoking (*n*/%)	38/33.3	30/36.6	0.637
Drinking (*n*/%)	53/46.5	37/45.1	0.849
Severity (*n*/%)	–	–	0.945
Mild	56/49.1	42/51.2	–
Moderate	53/46.5	34/41.5	–
Severe	5/4.4	6/7.3	–

AP, acute pancreatitis; BMI, body mass index; HDL-C, high density lipoprotein cholesterol; MetS, metabolic syndrome; RAP, recurrent acute pancreatitis.

### 3.2 Feature selection

**Figure 3:**
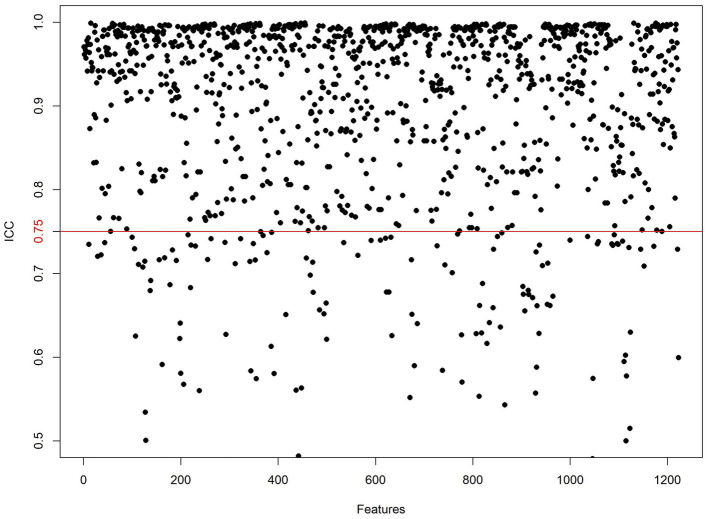
Inter-class consistency test. Values above the red line indicate ICC > 0.75, signifying high reliability of the radiomics features extracted by the two observers.

**Figure 4:**
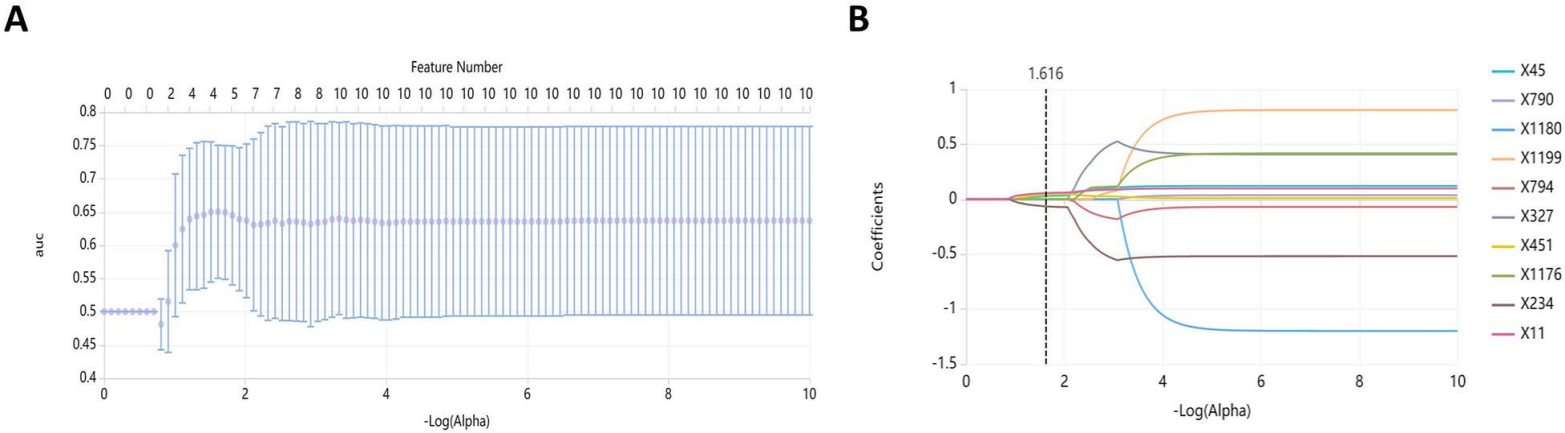
Feature dimension reduction using the least absolute shrinkage and selection operator (LASSO). **(A)** Feature selection; **(B)** Curve of coefficient variation.

**Figure 5:**
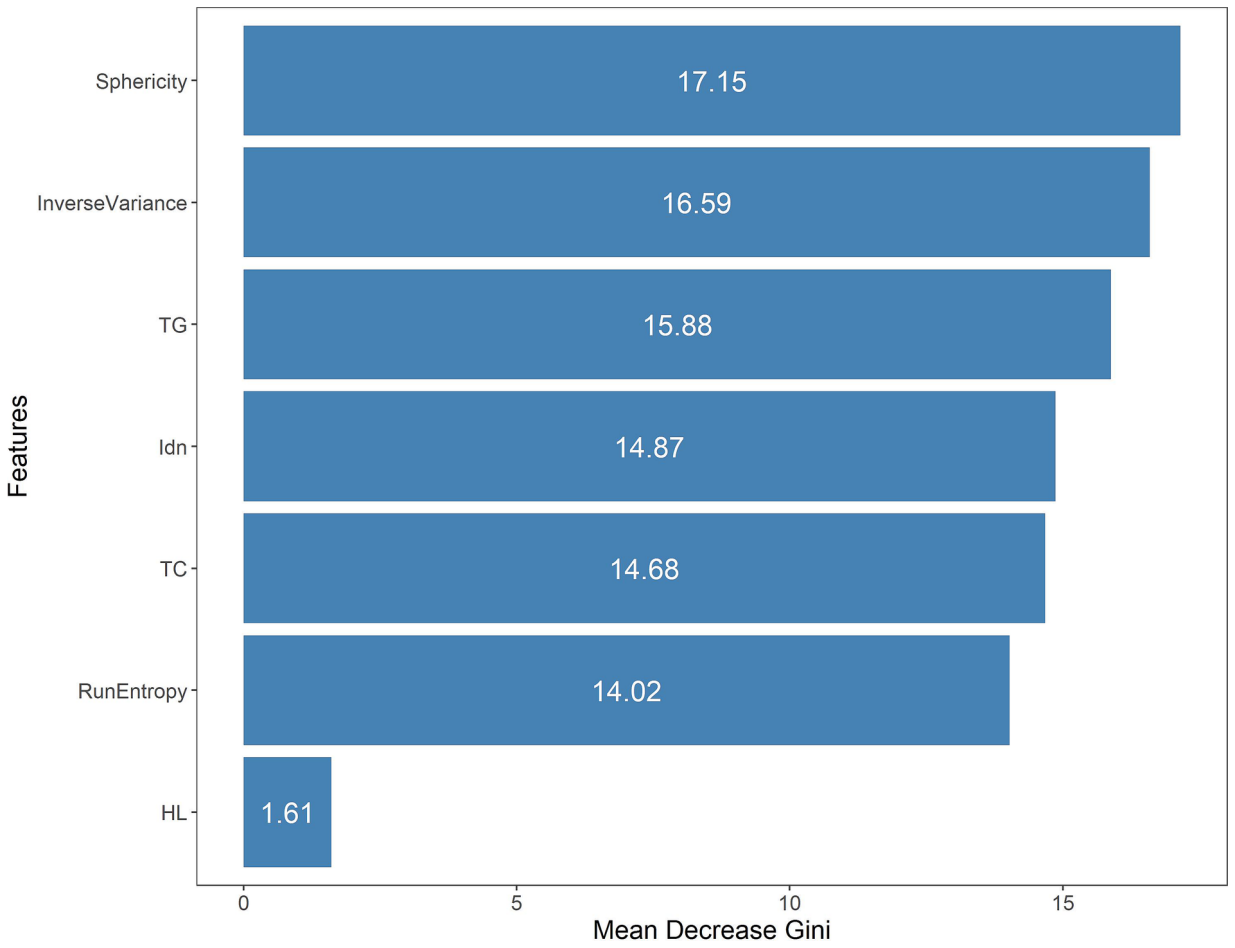
The seven features included in the combined model were ranked through mean Gini decrease value.

One thousand seventy features were retained when feature selection was performed with an ICC threshold > 0.75 (Figure 3). These included four optimal features chosen for model construction based on variance thresholding, K-best selection, and LASSO algorithm results (Figure 4). Random forest algorithm Gini coefficient means decrease-based rankings indicated that the most important radiomics feature was “Sphericity,” whereas triglycerides were the most highly ranked clinical feature (Figure 5).

### 3.3 Assessment of model performance

AUC of 0.883 (95% CI: 0.826–0.94), accuracy of 0.825, specificity of 0.875, and sensitivity of 0.754 were found in the combined model in the training group; AUC of 0.825 (95% CI: 0.752–0.897), accuracy of 0.766, specificity of 0.8, and sensitivity of 0.719 were found in the radiomics model; and AUC of 0.651 (95% CI: 0.562–0.74), accuracy of 0.664, specificity of 0.812, and sensitivity of 0.456 were found in the clinical model. In the test group, the combined model produced an AUC of 0.878 (95% CI: 0.791–0.964), the accuracy of 0.814, specificity of 0.882, and sensitivity of 0.72; and an AUC of 0.776 (95% CI: 0.656–0.897), the accuracy of 0.712, specificity of 0.794, and sensitivity of 0.6 for the radiomics model; and an AUC of 0.606 (95% CI: 0.472–0.74), the accuracy of 0.61, specificity of 0.824, and sensitivity of 0.32 for the clinical model (Table 2).

**Table 2:** Performance of three models in the training and test sets.

	Model	AUC (95%CI)	Specificity	Accuracy	Precision	Sensitivity
Training set	Combined model	0.883 (0.826–0.94)	0.875	0.825	0.811	0.754
	Radiomics model	0.825 (0.752–0.897)	0.8	0.766	0.719	0.719
	Clinical model	0.651 (0.562–0.74)	0.812	0.664	0.634	0.456
Test set	Combined model	0.878 (0.791–0.964)	0.882	0.814	0.818	0.72
	Radiomics model	0.776 (0.656–0.897)	0.794	0.712	0.682	0.6
	Clinical model	0.606 (0.472–0.74)	0.824	0.61	0.571	0.32

AUC, area under the curve; CI, confidence interval.

**Figure 6:**
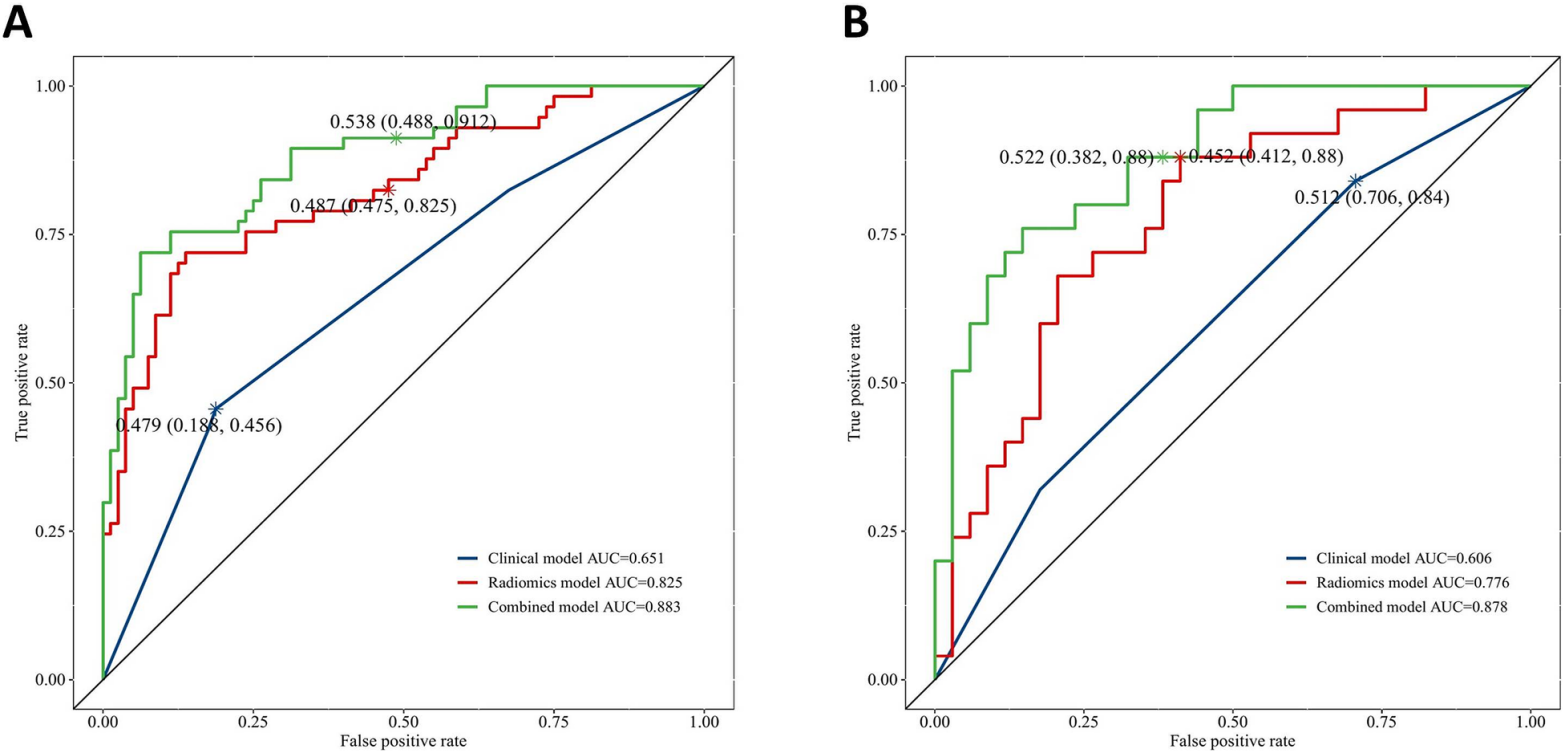
The receiver operating characteristic (ROC) curves for clinical model, radiomics model and combined model. **(A)** Training set; **(B)** Test set.

**Figure 7:**
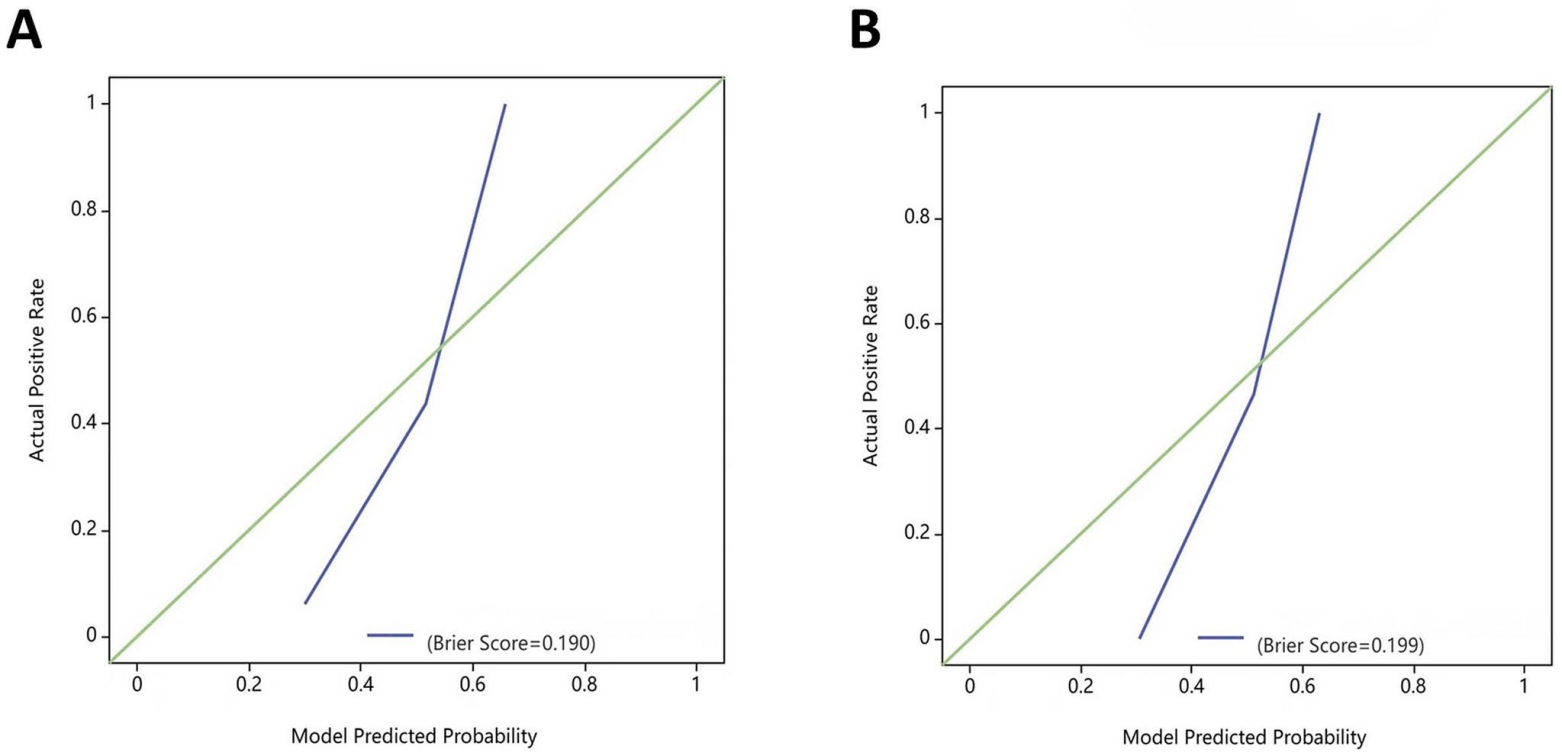
Calibration curves for the combined model in the training and test sets. **(A)** Training set; **(B)** Test set.

**Figure 8:**
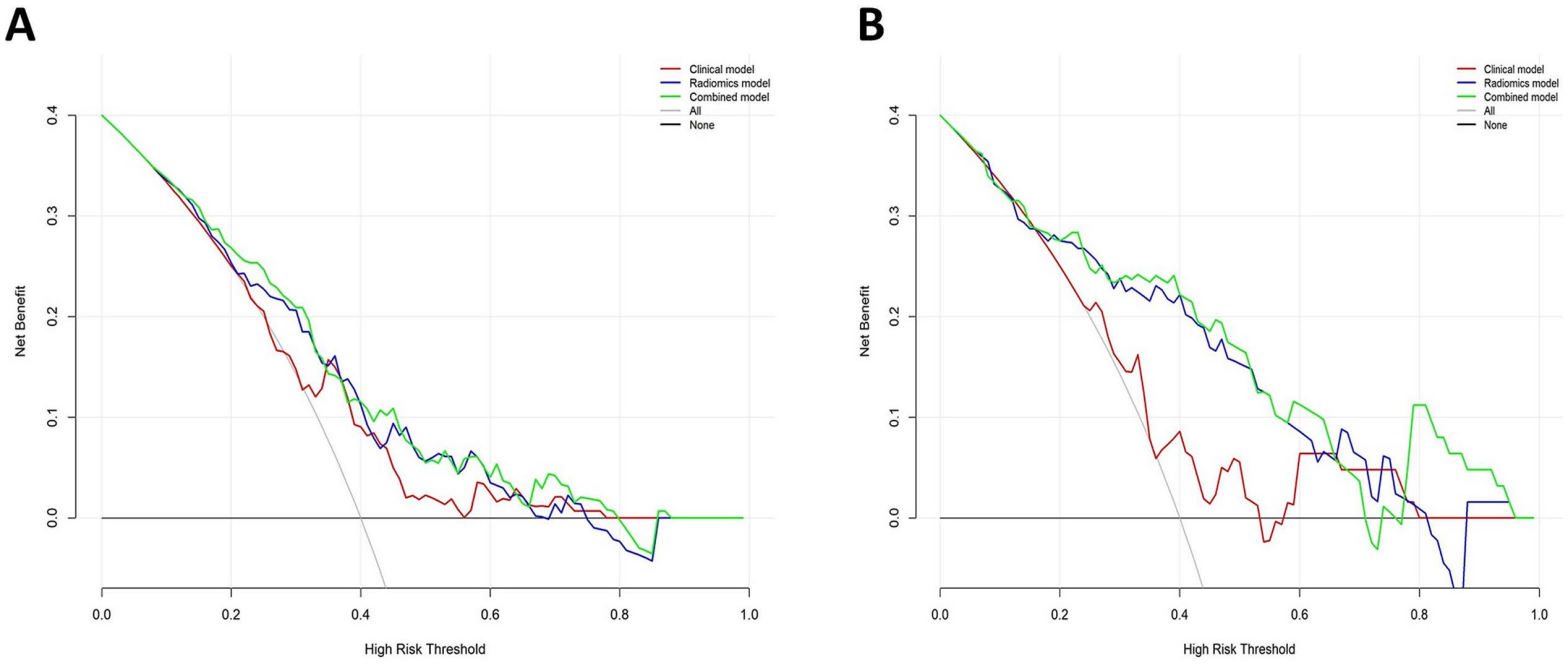
Decision curve analysis of three models. **(A)** Training set; **(B)** Test set.

The DeLong test confirmed that the combined model demonstrated significantly better predictive performance than both the radiomics (training set: *p* = 0.025, test set: *p* = 0.019) and clinical (training set: *p* < 0.001, test set: *p* < 0.001) models. The radiomics model consistently performed better than the clinical model (training set: *p* = 0.001, test set: *p* = 0.04) (Figure 6). In both the training and test sets, the combined model yielded the highest AUC, confirming its superior efficacy in RAP prediction. The clinical model alone had suboptimal performance, reinforcing the necessity of integrating radiomics features for improved diagnostic accuracy. Good consistency between predicted and actual recurrence rates was observed for both the training and test sets in the combined model when generating calibration curves, yielding respective Brier scores of 0.190 and 0.197 (Figure 7). DCA demonstrated that the combined and radiomics models outperformed the clinical model regarding the degree of clinical net benefit (Figure 8).

## 4 Discussion

As a chronic metabolic condition, MetS prevalence rates have progressively risen in recent years, with a parallel increase in the incidence of AP, which is an acute inflammatory disease. These trends have coincided with the recognition that AP patients with MetS often experience more severe disease and face a poorer AP prognosis (9, 30, 31). Previous studies have examined the correlation between MetS components and AP recurrence (17, 18). However, they predominantly relied on clinical data, and the available quantitative tools for assessing the likelihood of AP recurrence in patients with comorbid MetS have been insufficient. Thus, this study aimed to resolve this knowledge deficit by developing predictive models predicated on clinical characteristics and radiomics features, which provide a more detailed and quantitative assessment of disease-related features. The radiomics and combined models not only outperformed the clinical feature-based model in predicting RAP incidence among MetS patients but also demonstrated superior clinical utility, as indicated by DCA (32). Radiomics thus offers clear value as a source of clinical insight that can aid in formulating more effective individualized treatment strategies.

In the present research, 1,223 radiomics characteristics were acquired from T2WI MRI sequences of the subjects. A combination of variance thresholding, K-best selection, and the LASSO algorithm approaches ultimately led to the selection of four optimal features: Sphericity, Idn, Inverse Variance, and Run Entropy. Sphericity is a property that relates to lesion roundness and may reflect fibrosis-related morphological alterations. In contrast, Inverse Variance and Idn are GLCM features corresponding to spatial information about particular pairs of pixels with similar or specific intensity levels in an image, potentially linked to inflammatory infiltration (33). Run Entropy is a feature derived from the GLRLM corresponding to image texture variation complexity and uncertainty, which may correspond to microscopic architectural disruptions in RAP. These features provide a quantitative representation of pancreatic pathology, reinforcing their predictive value in radiomics-based assessment. Model construction was performed with a random forest classifier, and the included variables were analyzed with the Gini impurity-reduction feature-ranking technique, which ranks features by calculating the average reductions in Gini impurity when each variable is removed and comparing the results against corresponding Gini values for all other features (34). Sphericity was found to be the most important of the four best radiomics traits that were used in the development model. This may be related to the utility of Sphericity as a metric for assessing the shape of the region of interest. As repeated episodes of AP can lead to collagen deposition around ductal sites in the affected area, progressive acinar cell complex obstruction and consequent acinar atrophy can occur, leading to altered pancreatic morphology (35).

The radiomics model developed in this study may be better at making predictions for several reasons. Firstly, the start of AP might happen at the same time as the appearance of small changes in the pancreatic parenchymal tissue that cannot be seen with the naked eye (36, 37). Radiomics feature extraction, however, can detect these quantitative features that would otherwise be overlooked, leveraging them to establish new approaches to assessing the odds of AP recurrence (20). Secondly, the images in this study were derived from the same MRI instrument model with identical parameters, potentially limiting the effect of any variability in scanning parameters on extracted feature reproducibility, affording superior stability and repeatability (38, 39). Thirdly, the sequential implementation of the variance thresholding, K-best selection, and the LASSO algorithm approaches during feature selection also enabled removing redundant features while retaining highly reliable, relevant, and accurate features. The LASSO algorithm is widely used for regression analyses of high-dimensional datasets, and it is suited to use with small samples and a broad array of features such that the most relevant features associated with a given disease can be established (40–42). Finally, the random forest ensemble learning method employs myriad decision trees to achieve high levels of robustness and accuracy while reducing the odds of overfitting through its multi-decision tree voting mechanism (34). The radiomics model designed in this study thus exhibited good predictive performance, providing a novel means of managing RAP for patients with MetS.

The superior performance of the combined model can be attributed to the complementary nature of radiomics and clinical features. While radiomics captures microstructural variations in pancreatic tissue, clinical parameters such as triglyceride levels reflect systemic metabolic disturbances associated with disease recurrence. The integration of these two data sources enables a more comprehensive and precise prediction of RAP, allowing for early risk stratification and targeted interventions. These findings highlight the potential of a multi-modal approach to enhance diagnostic accuracy and guide personalized treatment strategies. In this study, the two patient groups presented significant differences in triglyceride levels, total cholesterol levels, and hyperlipidemia; triglyceride levels were independently associated with the risk of AP recurrence among those with MetS. This is consistent with prior evidence supporting a close link between triglycerides and the recurrence of AP (43–45). This effect may be related to the release of pancreatic lipase from the pancreatic vascular bed. After that, it can hydrolyze excess triglycerides circulating in the blood to produce FFAs. These, in turn, can injure platelets, microcirculatory endothelial cells, and acinar cells if they are not bound by albumin, resulting in the production of inflammatory mediators, the impairment of blood flow, and overall endothelial dysfunction (46, 47). Chylomicron levels in the blood also rise with levels of triglycerides, leading to elevated blood viscosity and altered pancreatic blood flow, culminating in ischemia and acidosis within the pancreas (47, 48). Active interventional strategies, including lifestyle changes, dietary adjustments, and appropriate pharmacological treatments, are thus warranted for patients with high levels of triglycerides to reduce the odds of RAP.

This study has some limitations. First, a major limitation of this study is the lack of external validation, as the data was collected from a single center. Future studies will need to include multi-center cohorts to validate the model’s generalizability and stability. Second, although the radiomics analyses in this study were based on a single T2WI MRI sequence that may not capture all relevant information about disease-related characteristics, the ultra-long TR scanning technique employed herein yielded images with an improved signal-to-noise ratio. Third, this study defines obesity as BMI ≥ 28 kg/m^2^, which aligns with the metabolic characteristics of the Chinese population. However, this definition may not fully reflect abdominal obesity as defined in the metabolic syndrome criteria, potentially limiting the generalizability of our findings to populations with different definitions of obesity. In the future, research efforts will focus on incorporating additional MRI sequences and expanding the patient cohort to develop more comprehensive and generalizable predictive models.

## 5 Conclusion

In summary, T2WI MRI sequence-derived radiomics features can provide insight into the intrinsic differences that characterize first-onset AP and RAP in individuals with MetS. The radiomics and combined models developed herein were capable of predicting the recurrence of AP more effectively among MetS patients as compared to the clinical model. Radiomics may thus hold value as a noninvasive and quantitative analytical strategy suitable for gaging the risk of AP recurrence when evaluating individuals with MetS, enabling physicians to formulate personalized treatment plans to optimize therapeutic approaches and achieve better patient outcomes.

## Data Availability

The original contributions presented in the study are included in the article/supplementary material, further inquiries can be directed to the corresponding author.

## Footnotes

^1^ https://www.slicer.org/

^2^ https://www.r-project.org/
